# Applying the Readiness for Interprofessional Learning Scale (RIPLS) to medical, veterinary and dual degree Master of Public Health (MPH) students at a private medical institution

**DOI:** 10.1371/journal.pone.0234462

**Published:** 2020-06-11

**Authors:** Rohini Roopnarine, Ellen Boeren

**Affiliations:** 1 Department of Pathobiology, School of Veterinary Medicine, Saint George’s University, Saint George’s, Grenada, West Indies; 2 School of Education, University of Glasgow, Glasgow, Scotland, United Kingdom; University of Lincoln, UNITED KINGDOM

## Abstract

**Purpose:**

With the emergence of zoonoses such as Ebola, many medical educators, have recommended the need for providing Interprofessional Education (IPE) as a pedagogical tool for familiarizing medical (MD) students with the framework of One Health (OH). This is important as students need to understand, the wider impacts of animal and environmental health factors on human health. IPE initiatives which typically incorporate the principles of OH, can provide MD and veterinary (DVM) students with a greater awareness of the role that animal diseases and climate change have on global health. However, negative attitudes to IPE have been reported as a key limitation to IPE implementation. The purpose of this paper is to examine the differences in readiness for interprofessional learning of medical and other allied human health professional students, including veterinarians and students undertaking dual degrees in combination with a Master of Public Health (MPH). Reflecting on Role Theory (RT) and Social Identity Theory (SIT), the paper aims to contribute to the understanding of differences in perceptions that exist between different types of health professionals.

**Methods:**

Students at a medical University enrolled in MD, DVM, DVM MPH and MD MPH programs, were invited to complete the standardized Readiness for Interprofessional Learning Scale (RIPLS), which consists of 19 Likert scale items measuring concepts relating to teamwork, professional identity and roles and responsibilities. A total of 364 students across the four programs took part. Descriptive and inferential statistical analyses were performed to assess differences between the programmes.

**Results:**

Results indicate that MD students score lower on the different RIPLS items compared to DVM, MD MPH and DVM MPH students. DVM and DVM MPH students are generally more positive about the need for teamwork, while MD MPH and DVM MPH students have a stronger positive identity about the need for IPE.

**Conclusions:**

The findings drawn from this study suggests that the MD students keep on seeing themselves as a separate group of health professionals in their own right. In order to guarantee an increased level of understanding on issues relating to the human-animal-environmental spectrum, medical curricula might benefit from the incorporation of shared learning and teamwork, as occurs within the MPH, enabling students to appreciate the value of interprofessional collaboration to their future practice. This is especially important during a time at which human-animal-environmental issues are affecting social and economic life worldwide.

## Introduction

The complexity of health care globally, has been a driving force for the implementation of Interprofessional Education (IPE) internationally [1]. Historically, IPE has been focused on collaboration between members of the medical and allied human health professions, often excluding veterinarians [2]. The emergence of diseases of animal origin transmissible to man such as Ebola and Coronavirus disease (COVID-19), suggests the need for an expanded cooperation and collaboration. Specifically, the inclusion of veterinarians into the collaboration will enhance the global efforts to address the health of the global community by identifying the aetiology more efficiently and reducing disease spread. The One Health (OH) concept provides such an approach for an inclusive collaborative networking between healthcare professionals working at the animal-human-environmental interface. Thus, attending to unique disease threats posed to global health welfare [3].

Unfortunately, the culture of the medical disciplines has long established siloes that have deterred collaboration between the disciplines and training [2]. Addressing global disease threats such as Ebola and COVID-19, requires a shift in the educational framework of health-related disciplines, but in particular veterinarians and physicians. IPE as a pedagogical tool for including the principles of collaborative practice as embodied by the OH concept, provides a framework for educating future graduates of each discipline for cooperative practice [4]. The success of IPE promoting an OH approach to practice requires students to be ready to engage in interprofessional learning. Negative attitudes towards collaborative practice are known to be a key inhibitor to the success of IPE and thus, evaluating the students’ readiness for IPE, is crucial for informing the development of IPE curriculum [5].

Most Master of Public Health (MPH) program curricula provide students with content knowledge on health policy formation, the relevance of OH, epidemiology, social and behavioural determinants of health and environmental health amongst other key topics. These content areas prepare dual degree students Doctor of Veterinary Medicine (DVM/MPH) and Doctor of Medicine (MD/MPH) for practicing collaboratively [6]. The MPH program at the institution where this research was conducted, has required competencies for students to demonstrate inter-professional values and communication skills that facilitate respect for different cultures, roles and responsibilities and the expertise represented by other professionals that work in global health.

In this paper, we focus on exploring the readiness of MD and DVM students as well as MD and DVM students who combine their study with an MPH degree for IPE using the Readiness for Interprofessional Learning Scale (RIPLS) [7]. As such, the research question we aim to answer in this paper is the following:

What are the differences in readiness scores for IPE between the medical, veterinary and dual degree programmes that follow different curricula?

This paper will start with a discussion of the previous literature on readiness for interprofessional learning and link these discussions to concepts of Role Theory (RT) and Social Identity Theory (SIT). It will then provide details on the methodological procedures undertaken as part of this research project, present and discuss the results [8]. The paper will review the results in light of the proposed theories and will formulate recommendations for practice and future research.

Readiness for Interprofessional Education has been discussed in the previous literature and has often been defined according to dimensions of the need for collaboration and teamwork among different health professionals, their roles and responsibilities, and their professional identity, values and beliefs. Twenty years ago, Parsell and Bligh [9] operationalised these concepts into a new empirical instrument, the Readiness for Interprofessional Learning Scale (RIPLS). While the academic community has explored readiness in various settings, the research on readiness for IPE presented in this paper is the first known attempt to compare the readiness of DVM and dual degree MPH students alongside MD students. This is important as more insights are needed on the further development of IPE that includes OH for a wider variety of medical professions, including veterinarians and those specialising in public health.

Examples of previous research include work by Sollami, Caricati, and Mancini [10] using the RIPLS reporting that medical students were the least ready for IPE. Morison et al. [11] reported that the RIPLS showed that medical students had a strong negative rather than positive professional identity with many students agreeing with the negative statements, indicating reluctance to engage in IPE. This is consistent with other studies [12], which suggest that students from high status groups, such as medicine, perceive their hierarchical supremacy above other groups. According to De Oliveira et al. [13] this may be explained by the confidence medical students hold about their roles, perceiving themselves as the key figures involved in patient care. This view is supported by Aziz, Teck, and Yen [14] who observe that MD students often assume they have more skills and knowledge to obtain than other groups. This attitude is a potential barrier to IPE development.

The interpretations highlighted in previous research can be linked to existing theories, more specifically, Role Theory (RT) [15] and Social Identity Theory (SIT) [16]. RT can be used to explain people’s behaviour as the result of a set of socially defined norms and expectations that are being assigned to a specific role. In the labour market, professionals will often have a specific perception about the ‘role’ they are carrying out. SIT claims that people’s identity is influenced by others who belong to the same group as they do. People carrying out a similar profession will likely conform to unwritten rules in their group and carry out their roles accordingly.

Roles and identities can thus be explored from a structuralist perspective that ties individual to group norms. As Merton et al. [15] discuss, the medical institution provides the social context that influences the development of the perceived professional roles and values the emerging medical graduate adopts, and this tends to differ between the human health professions versus the veterinarians. However, professional roles may alter as the structure of the social context alters [17]. For example, the expectations of the physician and veterinarian to engage in interprofessional roles may change upon the introduction of mandatory engagement with interprofessional learning and collaborative practice. This might then also change an individual’s social identity or consciousness of who they are as determined by their professional group, and the sense of alliance they perceive with the culture of that discipline. This is the core business of SIT. The students’ professional identity within the term readiness, includes the establishment of professional value concepts and moral standings in their careers [18]. Khalili, Orchard, Laschinger, and Farah [19] discuss that individual health programs inculcate student bias and distrust of other professional groups so that engagement in IPE was perceived as a threat to their identity. Sollami et al. [10] argues that historically, medicine has held a higher status and power over other groups, making it challenging for medical students to accede to situations where equal levels of power are ascribed to other groups as occurs in team settings and IPE.

Given the evidence of human medical professionals perceiving their distinct roles and identities as a fact, more thought needs to be given on how to prepare medical students to operate across human-animal-environment spheres as practitioners. Lewitt, Cross, Sheward and Beirne [20] in a recent article, express the view that early introduction of IPE coupled with social frameworks that are sensitive to professional identity frameworks are essential for interprofessional learning. According to Armitage-Chan and May [21] the temporal development of professional identity is consonant with the enunciations of Perry’s framework [22]. Perry [22] described the progressive cognitive development that occurs in students from the novice stages of enrolment, during which they hold singular perspectives, to a senior level, where they begin to view and value alternate perspectives. In this study presented in this paper, the lens of SIT will consider how MD or DVM students’ Professional identity is affected by the social context of their program, medicine or veterinary medicine, and relates to their readiness for IPE. In pedagogical terms, this need for IPE relates to Lave and Wenger‘s [23] Communities of Practice (CoP) framework in which a group of people share a similar concern and learn from each other to increase the effectiveness of their work. Applied to IPE, this refers to the need for the medical world to cooperate among the human-animal- environmental sphere.

## Materials and methods

### Location of the study

The study was conducted at a transnational private for-profit medical institution based offshore in the Caribbean. These types of institutions are unique Higher Education Institutions (HEI) that target the recruitment of predominantly North American students intending to return to medical and veterinary medical practice in North America [24]. This HEI offers programs that include the Doctor of Medicine (MD) and Doctor of Veterinary Medicine (DVM) with dual degree programs offered to MD and DVM students wishing to pursue a concurrent Master in Public Health (MPH). IPE is not offered within the core MD and DVM programs. However, dual degree MD/MPH and DVM/MPH are exposed to IPE through the shared classes they conduct with each other.

### Research design

The Readiness for Interprofessional Learning Scale (RIPLS) developed by Parsell and Bligh [9] was used to measure students’ readiness for IPE. The research population consisted of students from the medical University described above. The research population consisted of all MD, DVM and MD/MPH and DVM/MPH students that met the inclusion criteria for the study [25]. These inclusion criteria were that students had to have completed courses which included content in epidemiology, parasitology, microbiology, pathology, OH and in the Veterinary School, the course of veterinary public health, in order to have a basic understanding of the importance of the study. All of the dual degree MPH students met the inclusion criteria through their involvement in the MPH which in itself offers OH content and IPE through the shared classes that MD and DVM students participate in through this program. All 864 students across the programs meeting the inclusion criteria were invited to participate in the study. In total, 364 students (41%) participated in the research. This group consisted of 237 MD students, 78 DVM students, 145 MD/MPH students and 22 DVM/MPH students.

#### Instrumentation

RIPLS consists of 19 items measured through 5-point Likert scales. Original research by Parsell and Bligh [9], who constructed RIPLS as a way to test common conceptualisation of readiness in the literature, revealed a three-factor structure: teamwork and collaboration; professional identity and roles and responsibilities. Doubts regarding the reliability of this instrument, particularly the domains of professional identity and roles and responsibility, led to the formulation of a four-factor model by McFadyen et al. [7] that appeared far more reliable than the original three factor construct [9]. The four-factor construct represented the following dimensions: teamwork and collaboration, negative professional identity, positive professional identity and roles and responsibilities.

For the purpose of this research study, the original items were presented in the same order. In relation to McFadyen’s dimensions [7], these refer to: teamwork and collaboration (items 1–9), positive professional identity (items 13–16) and negative professional identity (items 10–12) and roles and responsibility (17–19). RIPLS was embedded in a questionnaire that gathered additional information on the demographics of the participants including their age, gender, ethnicity, nationality and program of enrolment.

Answers to the 19 items were given on 5-point Likert scales (1 = strongly disagree, 2 = Disagree, 3 = neutral, 4 = agree, and 5 = strongly agree). For items 1–9 and 13–16 representing the domains of teamwork and collaboration and positive professional identity respectively with higher scores indicating readiness for IPE. The answers for items 10–12 and 17–19 were given on 5-point Likert scales too and reverse scored as was done by McFadyen et al. [7]. (1 = Strongly agree, 2 = agree, 3 = neutral, 4 = disagree, 5 = Strongly disagree). Descriptive statistics for individual items will be presented using the original scaling. Higher scores for the reverse coded items reflect greater readiness towards IPE.

#### Data collection

Data collection was carried out using the Qualtrics platform (Qualtrics, Provo, UT). Initially, the questionnaire was piloted with a total of 20 students, five students from each of the four programs represented in this study. The questionnaire did not require modification as individuals that completed the pilot indicated that the statements were clearly understood across the groups. Once this was completed, the pilot data were removed from the Qualtrics (Qualtrics, Provo, UT).

All students meeting the inclusion criteria for the study were invited to participate. Students agreeing to participate were emailed a link to the survey. Students were sent weekly email reminders to encourage their participation. After eight weeks the hyperlink was disabled and data collection ended.

#### Data analysis

In total, 364 student respondents across the MD, DVM and Dual degree MD/MPH and DVM/MPH programs completed all of the closed ended survey items. The data were analysed using the Statistical Package for the Social Sciences (IBM SPSS version 24). We decided to conduct an Exploratory Factor Analysis using Principal Component Analysis (PCA) with a varimax rotation, to determine if conducting a robust data reduction technique on the 19 items in this study, would produce the same four factor construct obtained by McFadyen [7]. This was undertaken to help us interpret the data according to the dominant conceptualisation of ‘readiness’ in relation to teamwork and collaboration, positive professional identity, negative professional identity, and roles and responsibilities. The Kaiser-Meyer-Olkin (KMO) and Bartlett’s test of sphericity was used and confirmed the suitability of the data for factoral analysis. An evaluation of the loading together of items within key components enabled a hypothetical deduction to be made about the relationships between the items and the domains they represented. Factor loadings of 0.4 or greater were considered to be acceptable [26]. The reliability of the RIPLS was assessed using Cronbach’s alpha for each construct identified through PCA. According to Tavakol and Dennick [27] an α coefficient ≥0.70 indicates an acceptable reliability. EFA on the data collected from this study, produced a three factor construct that differed from McFadyen [7] as the items from the domains of Negative Professional Identity and Positive Professional identity clustered together to form one component, similar to the original factor structure presented by Parsell and Bligh [9]. The reliability of the three-factor identified in this study was compared to those domains of the four-factor construct [7] using Cronbach’s alpha, and are discussed below. Regardless of the factor structure, mean and standard deviations of all 19 Likert items are being presented below for students across the four programmes that took part in this study. Analysis of Variance has been conducted on each item to control for significant differences among groups. Given the small N for the DVM/MPH group, interpretations of the data on top of p-values are provided.

## Ethical considerations

IRB approval was obtained as human subjects were recruited for the study. The consent form was embedded within the survey on the Qualtrics platform, which enabled the respondents to read the form and provide their informed consent indicating their agreement to participate in the survey by selecting yes or no. The approval was obtained from both the St. George’s University Institutional Review Board (IRB) IRB Number: IRB00010095 where the research was conducted, and the Virtual Programs Research Ethics Committee (VPREC), the ethics committee at the University of Liverpool, UK-no ID number for this board approved this study. The participants were emailed a copy of the Participant information and consent forms for the study. They were then provided with a link to the survey on the Qualtrics platform. The consent form was embedded within the survey on the Qualtrics platform, which enabled the respondents to read the form and provide their informed consent indicating their agreement to participate in the survey by selecting yes or no.

## Results and discussion

Of 864 students across the MD, DVM and Dual degree MPH programs that were invited to participate in the study, 364 completed the 19 items on the RIPLS survey. Overall students across the disciplinary groups were positive about IPE.

### Factor analysis of the RIPLS

The KMO test (0.944) indicated a measure of sampling adequacy and Bartlett’s test of sphericity (p<0.05) indicated the RIPLS was suitable for factoral analysis. The PCA with a varimax rotation evaluated the validity of the four-factor model of McFadyen et al. [7] but generated a model that proposed a three-factor fit with Eigenvalues >1. PCA revealed three factors accounting for 67.337% of the total variance: 50% for factor 1 (teamwork and collaboration), 9.873% for factor 2 (identity) and 7.277% for factor 3 (roles and responsibility). The scree plot further supported the validity of the three-factor component model, with just three components having Eigenvalues greater than 1. Factor loadings of 0.4 or greater were considered to identify factor measured. The rotated factor solution using varimax rotation, provided in Table 1, showed that teamwork and collaboration (items 1–9) formed one factor. Items pertaining to negative identity (10–12) and positive identity (13–16) formed factor 2, similar to the overarching concept of “identity” as found by Parsell and Bligh [9]. The third factor, roles and responsibility, was composed of items 17–19. The findings of Spearman’s ranked correlation supported the findings of the factoral analysis. Items 1–9 and 13–16 were correlated with *r*_*s*_ ranging from 0.5 to 1, *p* = <0.05. Items 10–12 within the domain of negative professional identity correlated with items of positive professional identity (items 13–16) with *r*_*s*_ ranging between 0.5 and 1, *p* = <0.05, supporting the use of a single factor measuring identity. The items measuring roles and responsibility (17–19) were poorly correlated with other items.

**Table 1:** Rotated component Matrix-Varimax.

	Factors		
Item	Teamwork	ID	Roles
1	0.81		
2	0.86		
3	0.8		
4	0.76		
5	0.79		
6	0.8		
7	0.86		
8	0.85		
9	0.78		
10		-0.74	
11		-0.75	
12		-0.66	
13	0.52	0.66	
14		0.73	
15	0.47	0.7	
16	0.52	0.69	
17			0.76
18			0.55
19			0.72

### Reliability

#### Cronbach’s alpha

A reliability analysis was conducted on the three domains identified from the analysis of the three-factor construct identified in this study. Values for α were as follows; teamwork and collaboration (0.96); identity (0.89) and roles and responsibilities (0.48). Splitting identity in two sub-domains similar to McFadyen’s four factor solution [7], gave a Cronbach’s alpha’s of 0.79 for negative professional identity and 0.92 for positive professional identity. The domain of roles and responsibilities was found to be a poorly reliable construct in this study, as is consistent with the findings of McFadyen’s et al. [7], who found Cronbach’s alpha of 0.40 and 0.43 for two sets of data in this dimension. Parsell and Bligh [9] extracted roles and responsibilities of a third domain with Cronbach’s alpha of 0.32. Items were analysed separately as shown in Table 2 below as advised by others [12].

**Table 2:** RIPLS scores by individual items.

Item	MD	DVM	MD/MPH	DVM/MPH	F*	p-value
	n = 198	n = 68	n = 81	n = 17		
	Mean (SD)	Mean (SD)	Mean (SD)	Mean (SD)		
**TEAMWORK AND COLLABORATION**						
1. Learning with other students will help me become a more effective member of a health care team	4.08 (1.03)	4.38 (0.79)	4.35 (0.85)	4.53 (0.62)	3.23	.02
2. Patients would ultimately benefit if health care students worked together to solve patient problems	4.28 (1.00)	4.51 (0.61)	4.47 (0.87)	4.41 (0.80)	1.62	.18
3. Shared learning with other health care students will increase my ability to understand clinical problems	**4.11 (0.99)**	4.18 (0.83)	**4.46 (0.78)**	4.47 (0.62)	3.40	.02
4. Learning with health care students before qualification would improve relationships after qualification	4.09 (1.00)	4.34 (0.73)	4.33 (0.85)	4.53 (0.62)	2.88	.04
5. Communication skills should be learned with other health care students	4.21 (1.00)	4.44 (0.78)	4.36 (0.91)	4.65 (0.49)	2.02	.11
6. Shared learning will help me to think positively about other professionals	4.01 (1.00)	4.29 (0.75)	4.22 (0.99)	4.24 (0.90)	2.13	.10
7. For small group learning to work, students need to trust and respect each other	4.37 (0.94)	4.66 (0.51)	4.59 (0.79)	4.71 (0.59)	3.08	.03
8. Team-working skills are essential for all health care students to learn	**4.34 (0.95)**	**4.68 (0.53)**	4.47 (0.88)	4.53 (0.72)	2.64	.05
9. Shared learning will help me to understand my own limitations	4.07 (1.05)	4.16 (0.92)	4.32 (0.91)	4.41 (0.62)	1.64	.18
**NEGATIVE PROFESSIONAL IDENTITY**						
10. I don't want to waste my time learning with other health care students	3.75 (0.98)	3.96 (0.91)	3.94 (1.03)	3.94 (0.66)	1.20	.31
11. It is not necessary for undergraduate health care students to learn together	**3.74 (1.10)**	4.00 (0.99)	**4.10 (0.96)**	4.29 (0.69)	3.69	.01
12. Clinical problem-solving skills can only be learned with students from my own department	**3.84 (1.05)**	4.19 (0.90)	**4.37 (0.73)**	4.24 (0.83)	6.91	**< .001**
**POSITIVE PROFESSIONAL IDENTITY**						
13. Shared learning with other health care students will help me to communicate better with patients and other professionals	**3.96 (0.95)**	4.13 (0.93)	**4.31 (0.85)**	4.53 (0.51)	4.198	.01
14. I would welcome the opportunity to work on small-group projects with other health care students	3.69 (1.08)	3.87 (1.11)	3.99 (1.03)	4.00 (0.71)	1.89	.13
15. Shared learning will help to clarify the nature of patient problems	3.86 (0.98)	3.91 (1.08)	4.12 (1.03)	4.29 (0.59)	2.080	.10
16. Shared learning before qualification will help me become a better team worker	3.91 (0.96)	4.12 (0.87)	4.21 (0.90)	4.47 (0.62)	3.585	.01
**ROLES AND RESPONSIBILITIES**						
17. The function of nurses and therapists is mainly to provide support for doctors	3.42 (1.08)	3.47 (1.03)	3.70 (1.03)	3.71 (0.99)	1.57	.20
18. I'm not sure what my professional role will be	3.73 (1.01)	4.06 (0.87)	3.84 (1.05)	3.65 (1.22)	2.02	.11
19. I have to acquire much more knowledge and skills than other health care students	**2.66 (1.16)**	2.75 (1.14)	**3.09 (1.12)**	2.76 (1.15)	2.71	.05

* The degrees of freedom for the F statistics are df1 = 3, df2 = 360.

### Group comparisons

In order to answer our research question, we were interested in comparing groups of respondents according to their program of study. Given that comparisons needed to be made among four groups, Analysis of Variance (ANOVA) was conducted. Given the Likert scale nature of the data, normality assumptions were checked in order to justify this technique. Box plots, histograms and Q-Q plots and revealed the normal distribution of the data.

According to Royston [28] Shapiro and Wilk’s test are inaccurate for analysing large numbers of samples (larger than 50 samples) for normality and, hence, were not used in this study. Skewness and kurtosis measures were based on sample averages and not reported here as these measures are very sensitive to outliers leading to the impact of outliers being significantly accentuated [29]. All of the outliers were investigated to determine if they were valid measurements. They were found to be valid as they met the inclusion and exclusion criteria for the study and were, therefore, included in the study.

The Levene’s test was used to determine whether the assumption was met for equal variances for the comparisons being made for scores across the programs for the different Likert items.

The One-way ANOVA values were calculated for all 19 items and have been presented accordingly in its original order, representing underlying items for the constructs of teamwork and collaboration, negative professional identity, positive professional identity and roles and responsibilities. This structure will help in interpreting the data. Results are provided in Table 2 and consist of items’ mean, standard deviation, F and p-values. Significant group differences, using a Bonferroni adjustment, are indicated in bold. A warning is needed here as the small *n* for the DVM/MPH group limits the detection of significant effects in the data and the possibility for wider generalisations towards the research population. As such, an interpretation of raw scores is essential.

There was a statistically significant difference for item 12 at p < .001 level, ‘Clinical problem-solving skills can only be learned with students from my own department,’ which is part of the concept of Negative Professional Identity. On average, the MD students (mean = 3.84, sd = 1.05) scored significantly lower than the MD/MPH students (mean = 4.37, sd = 0.73). No other item yielded statistical significance at the p < .001 level but several other items were statistically significant at the p.<0.050 level.

Exploring the other results of this analysis revealed that MD students had lower mean scores on all 19 items of RIPLS compared to DVM, MD/MPH and DVM/MPH students. Nevertheless, all mean scores in relation to the teamwork and collaboration items are high, also for MD students, with the lowest score in this domain being 4.07 (sd = 1.07) for the statement ‘Shared learning will help me understand my own limitations.’ Interestingly, on seven out of nine items on teamwork and collaboration, DVM students scored higher than MD/MPH students, although the differences are very small. For six out of nine items, DVM/MPH students are the highest scoring group. Overall, the results indicate that teamwork and collaboration was perceived as more important among DVM and MPH students compared to students in the MD program.

Items in relation to negative professional identity are negatively correlated in RIPLS which might cause confusion among respondents. Results indicate that MD students scored lowest on both negative and positive professional identity. They are less convinced than students on other programmes that “shared learning with other health care students will help me to communicate better with patients and other professionals”, will help them to “become a better team worker” and “to clarify the nature of patient problems.” MD students are also more reluctant to undertake group work with students from other programmes.

Looking into the dimension of roles and responsibilities, DVM students are less sure about the professional role they will carry out compared to MDs. Especially MDs who combine their degree with an MPH feel they need to acquire more knowledge and skills than others. These results indicate that MD students in this study generally feel more comfortable with their future role expectations.

## Discussion

This, study started from the observation that despite the call by external stakeholders that collaborative practice is crucial for addressing current threats to global health, there is little evidence that this is a practice reality. To prepare future graduates for collaborative practice, it is important that they perceive and understand the relevance and value of collaboration. The results of this study indicated that MD students who have been exposed to IPE through the MPH demonstrated a greater readiness for shared learning. Additionally, DVM students tended to have slightly higher readiness than MD students. While all students showed high readiness scores across the different items, the differences between groups are worth a deeper investigation.

As highlighted in the introduction in this paper, Social Identity Theory (SIT) [16] and Role Theory (RT) [15] provide frameworks that show students’ readiness for shared learning might be influenced by their perception of their professional identity and their professional roles, underpinned by the cultural norms and expectations of their discipline.

These theories might thus mean that the socialization process of the dual degree students differs from that of the single degree programs, and that cultural differences exist between MD and DVM students. The exposure of IPE that occurs in the MPH program conveys that collaborative practice is an expectation of the future roles of the dual degree students, and it is therefore unsurprising that MPH students scored higher on positive professional identity items compared to those in single programmes.

Through the inclusion of the dual degree students in this study, these findings can be further explained by connecting the theoretical frameworks of role theory with SIT illustrating how this study expands upon the findings of others in the literature. Using the lens of SIT, the exposure to IPE/OH and the socialization that occurs in the MPH program seem to have broadened the dual degree student’s professional identity beyond the primary role of an MD or DVM. This view is consistent with those of others [10] who discuss that the lack of readiness especially among MD students for IPE, which may be due to the high status that society and the culture of medicine ascribe to this profession. Currently, there is no expectation by the institution for MD graduates to engage in interprofessional learning as IPE is a not mandatory requirement of the United States Medical Licensing Examinations (USMLE) for medicine.

SIT links the development of professional identity to readiness for IPE [30] which is linked to the students’ perceived relevance of IPE [31]. According to Armitage-Chan and May [21] as students’ progress through their degree programs, their readiness for IPE emerges through their ability to negotiate their identity to the demands of their context or program. This study expands on these previous findings through the inclusion of the MD/MPH and DVM/MPH student groups. The findings from this study confirm that medical and veterinary students that have been exposed to IPE through the MPH have more developed professional identities, which becomes a normalised part of their group characteristics to which they belong. The MPH program appears to provide a socialisation process for its graduates that breaks down the siloed cultures of the MD and DVM curricula that limit student readiness for shared learning.

A limitation of the study is the concerns expressed pertaining to the validity of use of the RIPLS for evaluating student readiness for IPE [32]. However, so far, the use of this instrument was found to be validated for use in this context for the following reasons. There are no ideal instruments for assessing the readiness of students for IPE [33]. In the absence of a more valid instrument, RIPLS is widely used in the literature for the purposes of evaluating student readiness for IPE and hence this study expands on an already well-developed evidence base. Parsell and Bligh [9] developed RIPLS as an empirical testing of the leading conceptualisation of ‘readiness’. The initial aim of our research was not to contest the concept of readiness, but to investigate whether there were differences in RIPLS scores between different groups of students at a medical University.

Despite issues raised about the psychometric capacity of the instrument soon after its development, Havier et al. [34] reported the instrument as one of the most appropriate for evaluating student readiness for IPE based on evidence-based support for the use of the RIPLS. Additionally, the Cronbach’s alpha scores for three of the four domains as constructed by McFadyen [7] confirm the reliability of the interpretation of concepts on teamwork and collaboration and negative and positive professional identity. Items of roles and responsibilities need detailed individual inspection because of its lack of conceptual consistency.

## Conclusion

The results of our study represent the first known report to compare the readiness of MD students alongside DVM, MD/MPH and DVM/MPH students for IPE. This study showed that MD students with the lowest RIPLS scores are the least ready for IPE, although their scores do not tend to be low. Our results suggest that across the DVM and dual degree programs, there is a less siloed way of thinking.

This study provided novel theoretical insight through the lenses of social identity theory and role theory for understanding the comparative differences in student’s readiness for IPE across the disciplines. The findings of this study provide evidence that conducting an MPH provides medical and veterinary students with greater readiness for IPE and collaborative practice.

The study illustrates the importance of introducing IPE to students to acculturate them to the importance of roles and responsibilities of the other professional group, the benefits of teamwork and collaboration for addressing the threats currently posed to global health. Specifically, institutions may want to engage in debates on how to incorporate more interprofessional curricula courses or research projects that involve MD and DVM students and faculty. The Community of Practice (CoP) model proposed by Lave and Wenger [23], and highlighted in the introduction section, can be used to bring the students, academic, and clinical faculty together across the disciplines to form an IPE community of practice. Institutions such as the one where this research was conducted, that offers medical, veterinary and, public health programmes on the same campus, are ideally positioned to develop IPE efforts through the development of campus communities of practice. Through these CoP’s, health professions students might become more familiar with the interprofessional culture which may promote the development of a professional identity that is more embracing of other groups [35]. IPE is more than a tool for providing content. The development of communication and teamworking skills and an appreciation of the roles and responsibilities of other professional groups is critical for executing collaborative practice as embodied by OH. Radical changes to the accreditation criteria of the medical disciplines, which might include a compulsory component of IPE, are unlikely to occur soon. To overcome this barrier, changes can be made using the University’s existing faculty resources to provide MD and DVM students with OH content independent of IPE. This is crucial for preparing the next generation of medical and veterinary graduates for engaging in collaborative practice towards address of the threats currently posed to health professionals working in the global environment.

## Supporting information

S1 Appendix RIPLS. (DOCX)
