# Supplementary material for: Applying the Readiness for Interprofessional Learning Scale (RIPLS) to medical, veterinary and dual degree Master of Public Health (MPH) students at a private medical institution
Source: PLoS One. 2020 Jun 11;15(6):e0234462. doi: 10.1371/journal.pone.0234462 (PMC7289424; doi:10.1371/journal.pone.0234462)
Supplement: S1 Appendix — (DOCX) [file pone.0234462.s001.docx]

# **S1 Appendix. Readiness for Interprofessional Learning Scale Survey**

Please indicate the following:

**Demographics**

**Age Ranges:** 18–24 ☐

25–34 ☐

> 35 ☐

**Gender:**

**Ethnicity:**

Black or African American ☐

Asian ☐

Native American ☐

Hispanic or Latino ☐

White ☐

Other ☐

**Nationality:**

US ☐

Canadian ☐

British ☐

European ☐

Asian ☐

Caribbean ☐

African ☐

Other ☐

**Professional program:**

MD ☐ MD/MPH ☐ DVM ☐ DVM /MPH ☐

**Program Term:**

**Prior Public Health Education/exposure: (MPH, participation in One Health clinics, other).** Please describe in the space below, if any**:**

**Are you familiar with the term “One Health”?**

Yes ☐ No ☐

**If ‘Yes’, please answer the following questions:**

1. Define the concept of One Health in the space below:
2. What is the relevance of the One Health concept to your practice as a global health professional? Please describe:

Please indicate the degree to which you agree or disagree with the statement by circling (**paper version survey**) the number of the response that best expresses your feeling.

**The scale utilised: 1=strongly disagree, 2=disagree, 3=neutral, 4=agree, 5=strongly agree.**

| 1. | Learning with other students will help me become a more effective member of a health care team | 1 | 2 | 3 | 4 | 5 |
| --- | --- | --- | --- | --- | --- | --- |
| 2. | Patients would ultimately benefit if health care students worked together to solve patient problems | 1 | 2 | 3 | 4 | 5 |
| 3. | Shared learning with other health care students will increase my ability to understand clinical problems | 1 | 2 | 3 | 4 | 5 |
| 4. | Learning with health care students before qualification would improve relationships after qualification | 1 | 2 | 3 | 4 | 5 |
| 5. | Communication skills should be learned with other health care students Good | 1 | 2 | 3 | 4 | 5 |
| 6. | Shared learning will help me to think positively about other professionals | 1 | 2 | 3 | 4 | 5 |
| 7. | For small group learning to work, students need to trust and respect each other | 1 | 2 | 3 | 4 | 5 |
| 8. | Team-working skills are essential for all health care students to learn | 1 | 2 | 3 | 4 | 5 |
| 9. | Shared learning will help me to understand my own limitations | 1 | 2 | 3 | 4 | 5 |
| 10. | I don't want to waste my time learning with other health care students | 1 | 2 | 3 | 4 | 5 |
| 11. | It is not necessary for undergraduate health care students to learn together | 1 | 2 | 3 | 4 | 5 |
| 12. | Clinical problem-solving skills can only be learned with students from my own department | 1 | 2 | 3 | 4 | 5 |
| 13. | Shared learning with other health care students will help me to communicate better with patients and other professionals | 1 | 2 | 3 | 4 | 5 |
| 14. | I would welcome the opportunity to work on small-group projects with other health care students | 1 | 2 | 3 | 4 | 5 |
| 15. | Shared learning will help to clarify the nature of patient problems | 1 | 2 | 3 | 4 | 5 |
| 16. | Shared learning before qualification will help me become a better team worker | 1 | 2 | 3 | 4 | 5 |
| 17. | The function of nurses and therapists is mainly to provide support for doctors | 1 | 2 | 3 | 4 | 5 |
| 18. | I'm not sure what my professional role will be | 1 | 2 | 3 | 4 | 5 |
| 19. | I have to acquire much more knowledge and skills than other health care students | 1 | 2 | 3 | 4 | 5 |

Sub-scales are:

SS1: Teamwork Co-operation – Items 1–9

SS2: Negative Professional Identity – Items 10–12 [reverse scored^*^]

SS3: Positive Professional Identity – Items 13–16

SS4: Roles & Responsibilities – Items 17–19 [reverse scored^*^]

Max/Min Scores are: 45/9; 15/3; 20/4; 15/3, respectively.

*items were reverse-scored to calculate the overall mean score, i.e., strongly disagree = 5, disagree = 4, neutral = 3, agree = 2 and strongly agree = 1.
